# The behavior of the electric potential across neuronal membranes of spinal ganglion and neuroblastoma cells

**DOI:** 10.1186/1471-2202-14-S1-P72

**Published:** 2013-07-08

**Authors:** Thiago M Pinto, Roseli S Wedemann, Célia M Cortez

**Affiliations:** 1 Instituto de Matemática e Estatística, Universidade do Estado do Rio de Janeiro, Rio de Janeiro, RJ, 20550-900, Brazil; 2 Science and Technology Research Institute, University of Hertfordshire, Hatfield, Herts, AL10 9AB, UK

## 

Electrical signals underlie the propagation of information in the nervous system. It is known that neuronal cells can generate electric potentials by diffusing ions across the neuronal membrane. We have previously studied the effects of electric charges fixed onto the inner surface of the membrane, on the potential of the membrane surfaces of healthy and cancerous neuronal cells [1,2]. Based on this work, we have developed a computational model that simulates the electric potential profile across neuronal membranes. This profile shows the behavior of the electric potential along the axis (*z*), perpendicular to the membrane, from the extracellular region to the inner cytoplasmic region. In particular, we compared the electric potential profile of the membranes of spinal ganglion and neuroblastoma cells, during the resting and action potential (AP) states. The spinal ganglion neurons represent healthy cells, while neuroblastomas denote tumorous neurons.

To analyze the electric potential profile of neuronal membranes, we numerically solved the Poisson-Boltzmann equation [1,3]. The model considers the following electric charges: (i) fixed on surfaces of the glycocalyx and the lypidic bilayer, (ii) dissolved in the electrolytic solutions for regions of the membrane model we have adopted, and (iii) fixed on the cytoplasmic proteins. All parameter values are based on measurements collected from experimental observations [4,5].

For the resting and AP states of spinal ganglion neurons and neuroblastoma cells, simulation results indicate that the electric potential significantly decreases along the *z *axis from the extracellular region to the surface of the glycocalyx. The decay of the potential is more expressive for the neuroblastoma than for the ganglion neuron. An interesting observation is that the electric potential continues to decrease across the glycocalyx region of the spinal ganglion neuron. This however does not occur for the neuroblastoma cells, whose potential does not change in this region of the membrane.

Because there is no electric charge within the lypidic bilayer, our results demonstrate linear variations of the potential across the bilayer of neuronal membranes. Furthermore, the intracellular potential of both spinal ganglion neurons and neuroblastoma cells exponentially increases from the inner membrane surface to the bulk cytoplasmic region during the resting state. However, during the AP state, the electric potential remains unchanged in the cytoplasm.

Our simulation results match those obtained for the membrane of the squid axon [3], whose mathematical model is based on similar ordinary differential equations to those of this study. Moreover, the different behavior of the electric potential observed in the spinal ganglion when compared to the neuroblastoma cells, in the glycocalyx region, may explain the difference in the electrophoretic behavior of these cells, as observed in experiments [4,5].
